# Computational Characterization of the Recently Synthesized Pristine and Porous 12-Atom-Wide Armchair Graphene Nanoribbon

**DOI:** 10.1021/acs.nanolett.5c01319

**Published:** 2025-05-07

**Authors:** Djardiel S. Gomes, Isaac M. Felix, Willian F. Radel, Alexandre C. Dias, Luiz A. Ribeiro Junior, Marcelo L. Pereira Junior

**Affiliations:** † Department of Applied Physics, State University of Campinas, Gleb Wataghin Institute of Physics, Campinas, São Paulo 13083-859, Brazil; ‡ University of Brasília, Faculty UnB Planaltina, 154624 Materials Science Postgraduate Program, Brasília, Federal District 70910-900 Brazil; ¶ Center for Agri-Food Science and Technology, Federal University of Campina Grande, Pombal, Paraíba, 58840-000 Brazil; § Physics Postgraduate Program, 28127 University of Brasília, Institute of Physics, Brasília, Federal District 70910-900, Brazil; ∥ Institute of Physics and International Center of Physics, University of Brasília, Brasília, Federal District 70910-900, Brazil; ⊥ Institute of Physics, University of Brasília, Brasília, Federal District 70910-900, Brazil; # Computational Materials Laboratory, University of Brasília, Brasília, Federal District 70910-900, Brazil; @ Department of Electrical Engineering, University of Brasília, College of Technology, Brasília, Federal District 70910-900, Brazil; △ Materials Science and NanoEngineering Department, Rice University, Houston, Texas 77005, United States

**Keywords:** graphene nanoribbons, porosity engineering, density functional theory, optical excitons, thermal transport, molecular dynamics

## Abstract

Recently synthesized porous 12-atom-wide armchair graphene nanoribbons (12-AGNRs) exhibit tunable properties through periodic porosity, enabling precise control over their electronic, optical, thermal, and mechanical behavior. This work presents a comprehensive theoretical characterization of pristine and porous 12-AGNRs based on density functional theory (DFT) and molecular dynamics simulations. DFT calculations reveal substantial electronic modifications, including band gap widening and the emergence of localized states. Analyzed within the Bethe–Salpeter equation framework, the optical properties highlight strong excitonic effects and significant absorption shifts. Thermal transport simulations indicate a pronounced reduction in conductivity due to enhanced phonon scattering at the nanopores. At the same time, MD-based mechanical analysis shows decreased stiffness and strength while maintaining the structural integrity. Despite these modifications, porous 12-AGNRs remain mechanically and thermally stable. These findings establish porosity engineering as a powerful strategy for tailoring graphene nanoribbons’ functional properties, reinforcing their potential for nanoelectronic, optoelectronic, and thermal management applications.

## Graphical abstract

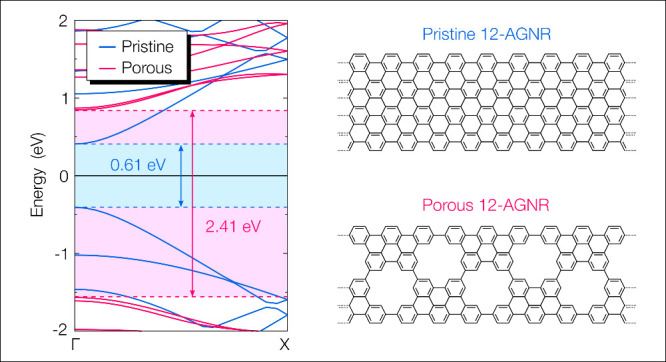

Graphene nanoribbons (GNRs) have garnered significant attention due to their distinctive electronic and structural properties, making them highly suitable for advanced electronic and optoelectronic applications.^1–5^ A key advantage of GNRs is their tunable band gap, which can be precisely engineered through width modulation, edge topology control, chemical functionalization, and periodic porosity introduction.^6–8^ These strategies have been extensively explored as practical approaches for tailoring electronic properties in low-dimensional materials.^9–11^

Recently, Fan et al. synthesized porous armchair graphene nanoribbons with a width of 12 atoms (porous 12-AGNR) on an Au(111) surface using a bottom-up surface-assisted reaction.^12^ Their experimental characterization, performed via scanning tunneling microscopy (STM) and scanning tunneling spectroscopy (STS), revealed a substantial band gap increase compared with pristine 12-AGNRs. Complementary density functional theory (DFT) calculations provided initial theoretical insights, yet several key physical properties remain unexplored.

In this work, we build upon these findings by providing a comprehensive theoretical characterization of pristine and porous 12-AGNRs. We investigate their energetic, thermal, and dynamic stabilities using advanced DFT-based calculations. Additionally, we refine previously reported electronic properties by employing the hybrid Heyd–Scuseria–Ernzerhof (HSE06) functional,^13,14^ which provides an accurate band gap estimation closer to experimental data. Optical properties are examined within the Bethe–Salpeter equation (BSE) formalism,^15^ incorporating significant excitonic effects. We employ fully atomistic classical molecular dynamics (MD) simulations to extend our analysis to larger scales to explore these nanoribbons’ thermal transport and mechanical properties.

To systematically evaluate the structural, stability, and electronic properties of these nanoribbons, for the first-principles calculations based on DFT, we used the Spanish Initiative for Electronic Simulations with Thousands of Atoms (SIESTA) code.^16–19^ Structural relaxations and preliminary electronic structure calculations employed the Perdew–Burke–Ernzerhof (PBE) generalized gradient approximation (GGA),^20,21^ widely recognized for its balance between computational efficiency and accurate description of atomic structure. To address the well-known band gap underestimation of GGA functionals,^22^ additional calculations were performed using the hybrid HSE06 functional^13,14^ via the HONPAS (Hefei order-*N* packages for *ab initio* simulations) package,^23,24^ which is entirely based on the SIESTA algorithm and numerical methods. We used norm-conserving Troullier–Martins pseudopotentials^25,26^ in Kleinman–Bylander form,^27^ with a kinetic energy cutoff of 500 Ry and a double-ζ polarized (DZP) basis set. Brillouin zone (BZ) integration was performed using a Monkhorst–Pack *k*-point grid of 10 × 1 × 1.^28^ Structural optimizations included complete relaxation of atomic positions and lattice vectors until residual atomic forces were below 0.001 eV/Å, with energy convergence set at 10^–5^ eV. Periodic boundary conditions were applied along the ribbon axis (*x*-direction), while vacuum regions of 35 Å (*y*-direction) and 50 Å (*z*-direction) minimized spurious interactions.

The optimized atomic structures of pristine and porous 12-AGNRs are shown in Figure 1. Porous 12-AGNRs were generated by selectively removing adjacent benzene rings along the ribbon axis, followed by the hydrogen passivation of the exposed carbon atoms. This procedure is illustrated in Figure 1(a). The final computational model adopted in this study is shown in Figure 1(b), where the unit cell consists of 84 atoms. To ensure a consistent comparison, all properties were also computed for pristine 12-AGNRs using a 3 × 1 × 1 supercell, yielding the same number of atoms as the porous structure.

**Figure 1:**
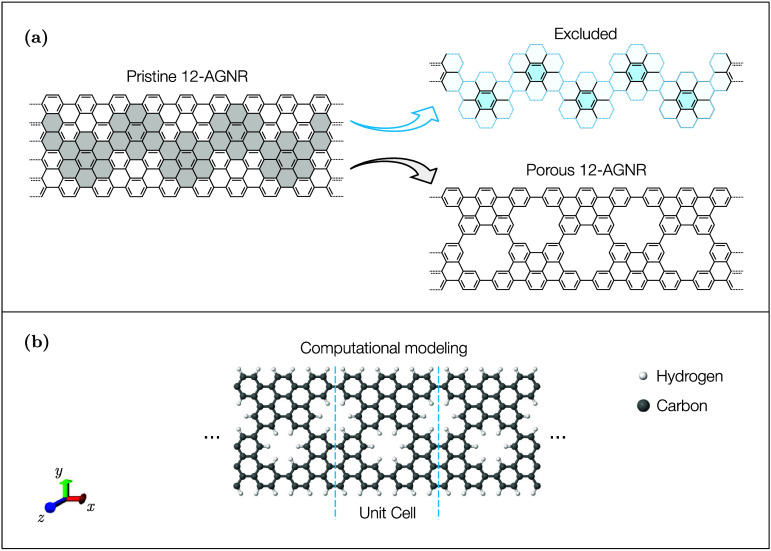
Atomic structures of the 12-AGNR models considered in this investigation. (a) Pristine and porous 12-AGNRs, where the porous structure is obtained by removing specific benzene rings and passivating the edges with hydrogen. (b) The computational model used in the simulations highlights the periodic unit cell. Carbon and hydrogen atoms are colored gray and white, respectively.

Pristine 12-AGNR exhibits C–C bond lengths ranging from 1.38 to 1.45 Å, with bonds parallel to the ribbon axis measuring approximately 1.44 Å at the center and 1.38 Å at the edges. In the porous 12-AGNR, bond lengths range from 1.38 Å (at the edges) to 1.50 Å (between consecutive pores), with increased variations around the pore regions. In both cases, C–H bonds remain at 1.10 Å and the structures retain *Pmmm* symmetry (Schoenflies notation: D_2h_-5). The lattice vector along the ribbon axis measures 12.97 Å for pristine 12-AGNR and 13.09 Å for porous 12-AGNR. Importantly, the overall morphology of the vacuum to isolate nanoribbons, including the periodic arrangement of pores, remains in good agreement with the experimental structure deposited on Au(111), except for minor deviations (below 2%) in selected bond lengths near pore edges.

Dynamic and thermal stabilities were further assessed. Phonon dispersion calculations were performed using a 3 × 1 × 1 supercell for porous 12-AGNR and a 9 × 1 × 1 supercell for pristine 12-AGNR, with an 800 Ry mesh cutoff. Energy and force convergence criteria were set at 10^–5^ eV and 0.04 eV/Å, respectively, with the acoustic sum rule enforced at the Γ point. The phonon dispersions (Figure S1) reveal that both systems are dynamically stable, as no imaginary frequencies appear. While the acoustic branches in pristine 12-AGNR are relatively flat, those in the porous counterpart exhibit increased dispersion due to the additional degrees of freedom introduced by porosity. In both cases, high-frequency modes around ∼3200 cm^–1^ correspond to C–H bond-length vibrations.

Thermal stability was examined via *ab initio* molecular dynamics (AIMD) simulations at 300 and 1000 K using a 168-atom supercell (6 × 1 × 1 for 12-AGNR and 2 × 1 × 1 for porous 12-AGNR) within the canonical (NVT) ensemble. A Nosé–Hoover thermostat^29^ controlled temperature, while a Parrinello–Rahman barostat^30^ regulated in-plane pressure. As shown in Figure S2, both systems maintained structural integrity under thermal conditions, with no significant atomic rearrangement. Additionally, these simulations provided estimates of the specific heat capacity, which remained comparable between pristine and porous 12-AGNRs, indicating that the porosity does not significantly affect this parameter. Since these nanoribbons have already been experimentally synthesized, our stability calculations further validate the accuracy and reliability of the computational methods employed in this study.

After confirmation of the structural and thermal stability, we analyzed the electronic properties of pristine and porous 12-AGNRs. Figure 2 presents the electronic band structures and projected density of states (PDOS), computed by using PBE and HSE06 functionals.

**Figure 2:**
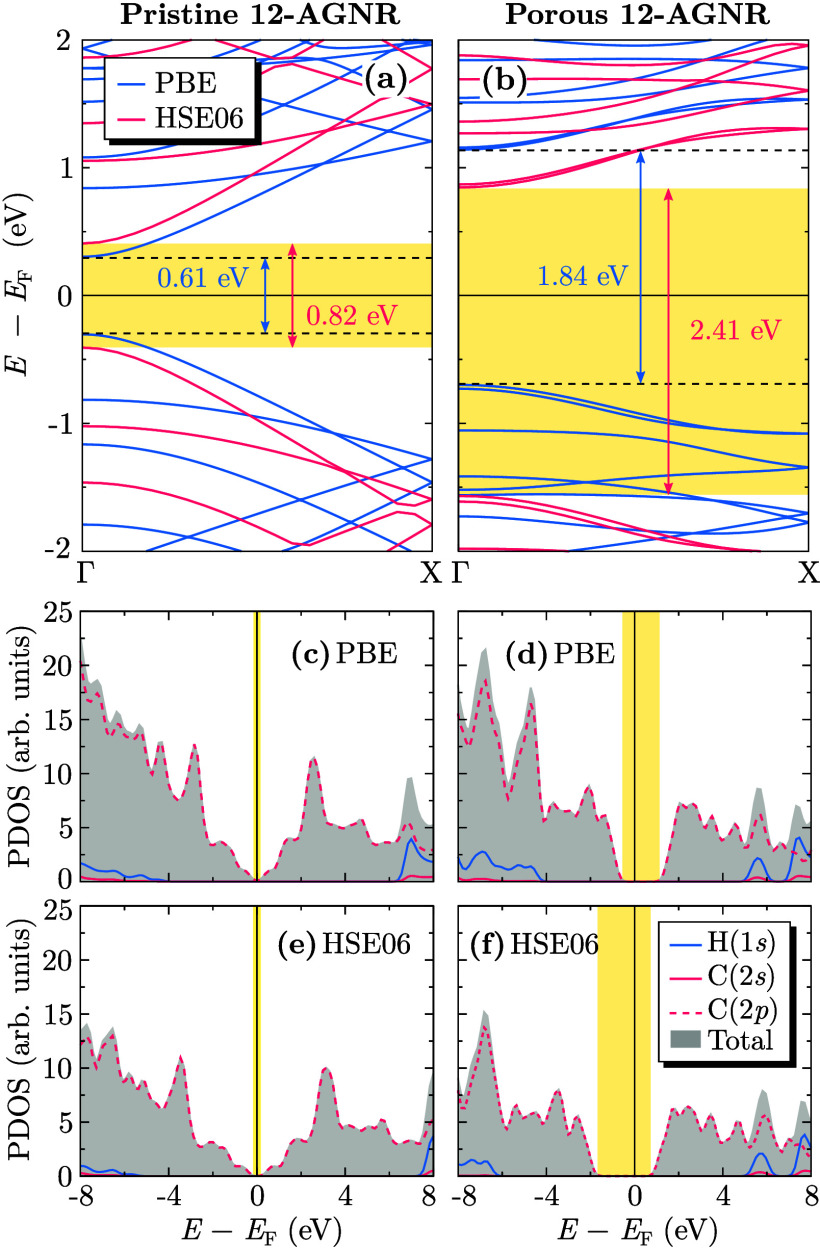
Electronic band structures and projected density of states (PDOS) of pristine and porous 12-AGNR nanoribbons. (a, b) Band structures computed using PBE (blue) and HSE06 (red) functionals, with shaded regions highlighting the band gap values. The numerical annotations indicate the energy gaps obtained for each functional. (c–f) PDOS for pristine (c, e) and porous (d, f) 12-AGNR nanoribbons, calculated using PBE (top row) and HSE06 (bottom row) functionals. The contributions of hydrogen (1s) and carbon (2s and 2p) orbitals are shown separately, with the total density of states represented by the shaded gray region. The yellow-lighted areas indicate the band gap region.

For pristine 12-AGNR (Figure 2(a)), a direct band gap of 0.61 and 0.82 eV was obtained with PBE and HSE06, respectively, with the conduction band minimum (CBM) and valence band maximum (VBM) located at the Γ point. These results align with previous theoretical calculations,^12^ where a gap of ∼0.6 eV was estimated using GGA-PBE. As expected, HSE06 predicts a larger band gap due to its improved treatment of exchange interactions. Experimental STS measurements reported a band gap of 1.13 eV for pristine 12-AGNRs on Au(111) substrates.^31^

Introducing periodic porosity (Figure 2(b)) substantially modifies the electronic structure. The band gap remains direct but increases to 1.84 eV (PBE) and 2.41 eV (HSE06), attributed to charge redistribution induced by periodic pore formation. This widening is consistent with experimental reports,^12^ where STS measurements indicated a gap of ∼3.3 eV for porous 12-AGNRs on Au(111). Theoretical estimates from the synthesis study predicted a band gap of 1.9 eV, closely matching our PBE results, whereas our HSE06 calculations better approximated the experimental values. The larger gap observed in the experimental STS data can be attributed to the dielectric screening effect of the Au(111) substrate and the structural modifications generated by the interaction of the monolayer with the substrate, which modifies the quasiparticle energies and enhances many-body interactions, leading to an increased measured gap.^32^

Beyond the band gap expansion, the conduction band in porous 12-AGNR exhibits flatter electronic states relative to its pristine counterpart, suggesting enhanced charge carrier localization due to periodic pore distribution. These features can impact electronic transport by reducing the mobility. This effect has been experimentally attributed to interactions between pore edges and the substrate.^12,31^ Furthermore, theoretical studies indicate that the electronic structure of porous 12-AGNR strongly resembles that of 3-AGNRs, implying that these subunits dominate its electronic behavior. Moreover, porosity significantly alters the effective masses, with the porous nanoribbons showing increased effective masses for electrons (0.1516) and holes (0.1796) compared to the pristine structure, which shows 0.0585 for electrons and 0.0581 for holes. The Supporting Information presents detailed information on calculating the electron and hole effective masses.

Hydrogen passivation at the ribbon edges and pore boundaries ensures electronic stabilization, preventing the formation of metallic or highly reactive states from unsaturated carbon atoms. Consequently, the observed electronic features arise intrinsically from periodic porosity rather than structural defects.

These findings are further supported by the PDOS analysis (Figure 2(c–f)). As expected, the electronic behavior near the Fermi level is predominantly governed by carbon 2p orbitals in both the valence and conduction bands, reinforcing the π character of the electronic states. Notable differences emerge between the two nanoribbon configurations. In pristine 12-AGNRs (Figure 2(c,e)), states near the Fermi level exhibit a relatively continuous distribution, indicative of a homogeneous electronic structure. Conversely, for porous 12-AGNRs (Figure 2(d,f)), a pronounced increase in the separation of electronic states is observed, consistent with the previously discussed band gap widening. Additionally, modifications in the conduction band density of states further support localized electronic states induced by periodic porosity.

The substantial band gap modulation caused by porosity suggests a significant impact on the optical properties of these nanoribbons, particularly in excitonic effects and absorption spectra. To capture these many-body interactions, we employ the BSE^15^ formalism to describe electron–hole interactions. It provides a detailed characterization of excitonic states and their contributions to the optical response.

Excitonic and optical properties were computed using the WanTiBEXOS code^33^ within the independent-particle approximation (IPA) and the BSE framework. The Supporting Information presents detailed information on calculating optical properties. A 25 × 1 × 1 **k**-mesh was adopted, considering 14 conduction and valence bands for pristine 12-AGNRs and 10 conduction and 8 valence bands for porous 12-AGNRs. However, direct interface compatibility with the SIESTA/HONPAS Hamiltonian is not publicly available but can be provided upon request. A detailed description of the BSE implementation, including the excitonic Hamiltonian formulation and computational parameters, is provided in the Supporting Information.

Figure 3 presents the linear optical response in terms of reflectivity, refractive index, and absorption coefficient, obtained within the IPA (pink curves) and BSE (blue curves) for pristine (left panels) and porous (right panels) 12-AGNRs. The incident light was considered to be polarized along the longitudinal axis of the nanoribbon. A pronounced redshift in the optical band gap is observed in Figure 3(a,b), leading to estimated exciton binding energies of 400 meV for pristine and 787 meV for porous 12-AGNRs. These values exceed those reported for two-dimensional carbon allotropes,^34^ a consequence of the enhanced quantum confinement in quasi-1D systems. Moreover, AGNRs of similar widths, exciton binding energies ranging from 300 to 600 meV, have been reported using GW+BSE methods.^35^ In narrower or more confined systems, such as quantum dots or ultranarrow GNRs, values exceeding 700 meV have been observed.^36^ Both structures exhibit absorption across the infrared (IR), visible, and ultraviolet (UV) regions with higher absorption coefficients at shorter wavelengths.

**Figure 3:**
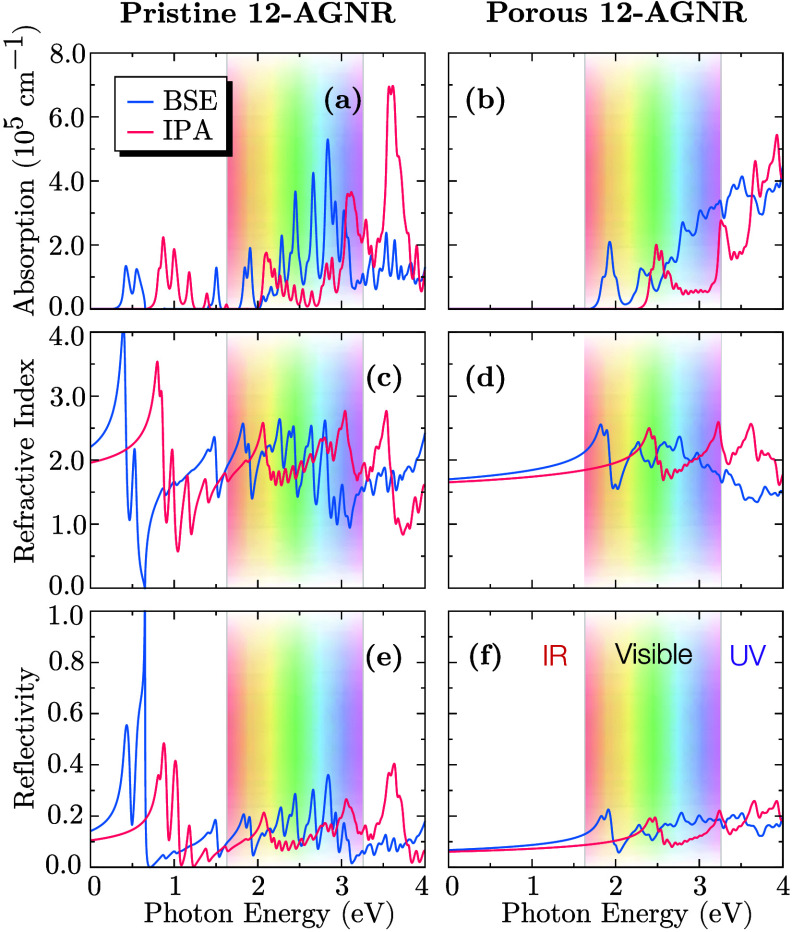
Optical properties of pristine (left) and porous (right) 12-AGNRs. (a,b) Optical absorption coefficient, (c,d) refractive index, and (e,f) reflectivity as a function of photon energy.

Pristine 12-AGNRs exhibit a stronger linear optical response than their porous counterparts, with a higher refractive index and reflectivity. Notably, total reflection is achieved for photons in the infrared spectrum, with partial reflection extending into the visible and ultraviolet regions. In contrast, porous 12-AGNRs display minimal reflection, limited to a narrow range in the visible and UV regions. These findings highlight the influence of periodic porosity on optical properties, reinforcing its role in tailoring the light–matter interaction in graphene-based nanostructures.

Large-scale atomic systems were considered to characterize the thermal transport and mechanical response of pristine and porous 12-AGNRs, as a fully quantum-mechanical treatment would be computationally prohibitive. To accurately capture long-wavelength phonon transport and mechanical response, supercells containing between 336 and 13440 atoms were employed, reaching lengths of up to 200 nm. Given these system sizes, we utilized classical MD simulations, which enable the investigation of atomic-scale dynamics at spatial and temporal scales inaccessible to first-principles methods. In classical MD, atoms are modeled as classical particles governed by Newton’s equations:

1 $$m_{i}\frac{d^{2}r_{i}}{dt^{2}}=-\frac{\partial E}{\partial r_{i}}$$

where *m*_*i*_ and **r**_*i*_ denote the mass and position of atom *i*, respectively, and *E* is the total potential energy. The accuracy of the computed properties depends directly on the choice of the interatomic potential. Here, atomic interactions were described using the second-generation reactive empirical bond order (REBO) potential,^37^ widely validated for carbon-based nanostructures.^38–43^ We note that recent developments in machine learning interatomic potentials (MLIP),^44,45^ such as the moment tensor potential (MTP), have shown promise for accurate thermal transport modeling in low-dimensional materials. All MD simulations were performed using the Large-scale Atomic/Molecular Massively Parallel Simulator (LAMMPS),^46^ with equations of motion integrated via the velocity-Verlet algorithm.^47^

Thermal transport properties were investigated using the reverse nonequilibrium molecular dynamics (RNEMD) method proposed by Müller-Plathe.^48^ Periodic boundary conditions were imposed along the longitudinal (*x*-axis) direction, and nanoribbons up to 200 nm in length (13,440 atoms) were partitioned into 20 slabs. Heat flux was induced by exchanging kinetic energy between high-velocity atoms at the extremities and low-velocity atoms at the center. After equilibration, a stable temperature gradient was established, enabling the calculation of the lattice thermal conductivity κ­(*L*_*x*_) as

2 $$\kappa \left(L_{x}\right)=-\frac{1}{\nabla_{x}T}\left[\frac{\underset{\mathrm{swaps}}{\sum }\Delta K}{2A\Delta t}\right]$$

where *A* is the cross-sectional area, given by the product of the ribbon width (∼1.5 nm) and thickness (*h* = 0.335 nm). The thermal conductivity follows a ballistic-to-diffusive length dependence, modeled as

3 $$\frac{1}{\kappa \left(L_{x}\right)}=\frac{1}{\kappa_{\infty }}\left(1+\frac{\Lambda }{L_{x}}\right)$$

where κ_∞_ is the intrinsic thermal conductivity and Λ is the effective phonon mean free path. Simulations were performed with a time step of 0.5 fs, with kinetic energy swaps every 500 timesteps, over a total duration of 20 ns.

Figure 4 presents the dependence of thermal conductivity on nanoribbon length with pristine 12-AGNRs shown for comparison. Symbols represent values computed via eq 2, while solid lines correspond to least-squares fits using eq 3. Independent simulations with varying initial atomic velocities were conducted to estimate uncertainties, yielding an uncertainty below 5% in all cases. Fluctuations in heat flux and temperature gradient were also considered, confirming a similar uncertainty level. The agreement between fitted curves and computed data highlights the predictive capability of eq 3, enabling the estimation of intrinsic thermal conductivity from relatively short simulations.

**Figure 4:**
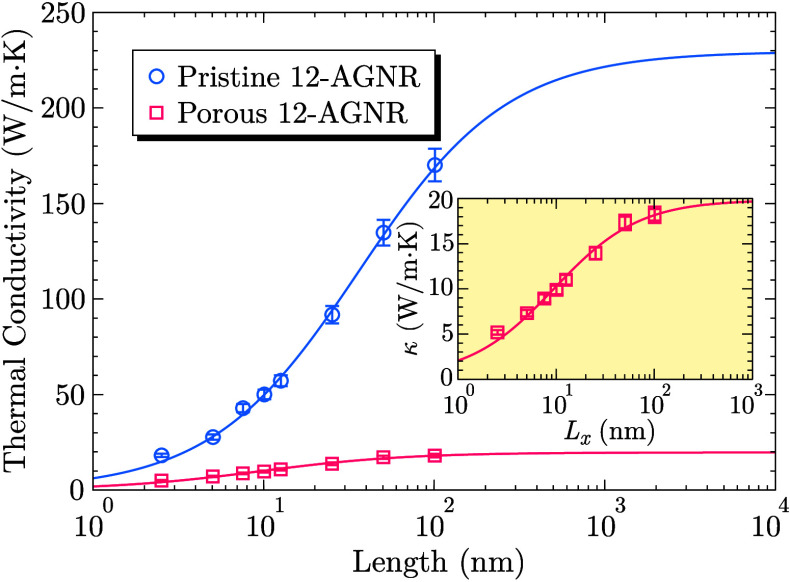
Thermal conductivity of pristine and porous 12-AGNRs as a function of the sample length. Data points correspond to RNEMD simulations, while solid lines are fitted by using eq 3. The inset shows the thermal conductivity values for the porous 12-AGNR plotted on a logarithmic scale, along with the corresponding fit, highlighting the saturation behavior at larger lengths.

The intrinsic thermal conductivity of pristine 12-AGNRs was 230 ± 22 W/m·K, in agreement with previous reports.^41,49^ As expected, porosity significantly reduces the thermal conductivity, yielding an estimated 19.7 ± 1.7 W/m·K. The dependence of thermal transport on pore density, size, and spatial distribution is well documented,^50–53^ with our results indicating a ∼90% reduction in κ compared to pristine 12-AGNRs. This decline is comparable to that observed in GNRs with isotopic doping,^42^ chemisorption functionalization,^43^ and Stone–Thrower–Wales defects,^54^ though less pronounced than in kirigami-engineered GNRs, where κ is reduced by approximately 2 orders of magnitude.^55^

Beyond thermal transport, structural modifications, such as porosity and edge morphology, also influence mechanical properties. We performed uniaxial tensile deformation simulations along the longitudinal direction to assess these effects. Given the computational demands of such analyses, shorter nanoribbons of approximately 50 nm (3276 atoms) were used. For statistical robustness, 10 independent simulations were conducted for each configuration at room temperature, varying initial atomic velocity distributions. Each system was equilibrated for 100 ps at 300 K before applying a continuous uniaxial deformation over 5 ns at a constant strain rate of 10^–4^ ps^–1^. The velocity-Verlet algorithm was employed for numerical integration, with a time step of 0.1 fs.

Atomic-level stress components were computed via the virial theorem and normalized by the inverse of the effective volume *V*:

4 $$\sigma_{\alpha \beta }=-\frac{1}{V}\left[\underset{i}{\sum }m_{i}v_{i\alpha }v_{i\beta }+\underset{i,j}{\sum }f_{ij\alpha }r_{ij\beta }\right]$$

where *m*_*i*_ is the mass of atom *i*, *v*_*i*α_ and *v*_*i*β_ are velocity components, *f*_*ij*α_ is the interatomic force between atoms *i* and *j*, and *r*_*ij*β_ is the displacement between them. The first term accounts for kinetic contributions, while the second represents interatomic interactions.^56,57^ The effective volume is computed as *V* = *L*_*x*_ × *W* × *h*, where *L*_*x*_ is the ribbon length, *W* is the previously mentioned width of approximately 1.5 nm, and *h* is the thickness.

The uniaxial strain was defined as ε = Δ*L*/*L*_0_, where *L*_0_ is the initial length and Δ*L* is the corresponding change upon deformation. In the elastic regime, stress follows Hooke’s law, σ = *Y*_M_ε, where *Y*_M_ is Young’s modulus. Stress–strain curves were computed consistently with the ribbon thickness (0.335 nm) adopted in the thermal transport analysis, ensuring direct comparability between the thermal and mechanical properties.

Figure 5 presents the stress–strain behavior of pristine and porous 12-AGNRs under a uniaxial tensile loading. In the elastic regime, stress increases linearly with strain, allowing for the extraction of *Y*_M_ by fitting the data to Hooke’s law. Pristine 12-AGNRs exhibit a *Y*_M_ of 784 ± 9 GPa, while porous 12-AGNRs display a 428 ± 3 GPa reduced modulus. These values align well with DFT calculations, which yield *Y*_M_ = 864 and *Y*_M_ = 494 GPa for pristine and porous 12-AGNRs, respectively. A slight overestimation in DFT results is expected, as finite-temperature effects inherent to MD simulations are absent in static DFT calculations, reinforcing the reliability of MD for mechanical property predictions. The observed ∼45% reduction in Young’s modulus reflects the weakening of the carbon network due to periodic pore formation, which disrupts the delocalized π-bonding responsible for the high stiffness of graphene-based materials.

**Figure 5:**
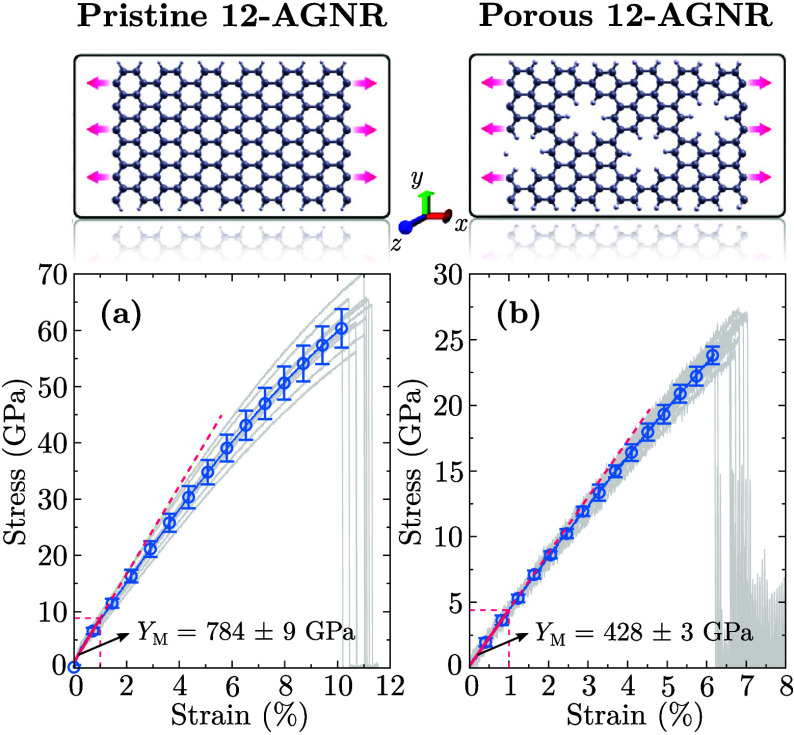
Stress–strain response of pristine (a) and porous (b) 12-AGNR nanoribbons under uniaxial tensile deformation.

In addition to reducing stiffness, the porosity also weakens the mechanical strength of nanoribbons, leading to lower critical strain and stress values. For pristine 12-AGNRs, the critical stress and strain are σ_C_ = 63 ± 4 GPa and ε_C_ = 10.9 ± 0.4%, respectively. In porous 12-AGNRs, these values drop to σ_C_ = 25 ± 1 GPa and ε_C_ = 6.7 ± 0.3%, demonstrating the direct impact of periodic voids on fracture resistance. The fracture mechanism is brittle in all cases with abrupt failure and no significant plastic deformation. Such a fracture mechanism is well-documented in the literature on graphene-based nanostructures.^58^ These results indicate that periodic porosity is an effective strategy for modulating the mechanical response of graphene nanoribbons while maintaining their structural integrity. The Supporting Information presents the MD snapshots for the Von Mises stress illustrating the fracture mechanism and the role of local stress accumulation.

Beyond mechanical properties, periodic porosity enables a highly tunable platform, where electronic, optical, and thermal properties can be systematically engineered.

In summary, we have conducted a comprehensive theoretical investigation of pristine and porous 12-AGNRs, a recently synthesized graphene nanoribbon system, analyzing their structural, electronic, optical, thermal, and mechanical properties. First-principles calculations revealed that periodic porosity significantly modifies the electronic structure, widening the band gap and introducing localized states. Optical properties demonstrated strong excitonic effects, resulting in a significant red shift in the optical band gap, emphasizing the role of many-body interactions. Thermal transport simulations showed a substantial reduction in conductivity due to phonon scattering at nanopores, while mechanical analysis confirmed a decrease in stiffness and strength with retained structural stability. These findings establish porosity engineering as a versatile tool for tailoring the functional properties of graphene nanoribbons, reinforcing their potential for nanoelectronic, optoelectronic, and thermal management applications.

## Supplementary Material



## Notes

Phonon dispersion relations confirming the dynamic stability of pristine and porous 12-AGNRs and *ab initio* molecular dynamics simulations at 300 and 1000 K validating their thermal stability; a comprehensive description of the Bethe–Salpeter equation (BSE) formalism, including the excitonic Hamiltonian, Coulomb interaction terms, and expressions for oscillator strengths; details on the effective mass calculation and atomic stress analysis; and figures including phonon spectra, AIMD energy profiles, dielectric functions from BSE and IPA approaches, and von Mises stress maps highlighting fracture patterns (PDF)
