# Supplementary material for: Computational Characterization of the Recently Synthesized Pristine and Porous 12-Atom-Wide Armchair Graphene Nanoribbon
Source: Nano Lett. 2025 May 7;25(21):8596–603. doi: 10.1021/acs.nanolett.5c01319 (PMC12123677; doi:10.1021/acs.nanolett.5c01319)
Supplement: Supplementary file 1 [file nl5c01319_si_001.pdf]

Supporting Information: Computational Characterization of the Recently Synthesized Pristine and Porous 12-Atom-Wide Armchair Graphene Nanoribbon

Djardiel da S. Gomes,[†,‡] Isaac M. Felix,[¶] Willian F. Radel,[§] Alexandre C. Dias,[‖,§] Luiz A. Ribeiro Junior,[⊥,§,#] and Marcelo L. Pereira Junior*,[@,§,△]

†*State University of Campinas, Gleb Wataghin Institute of Physics, Department of Applied Physics, 13083-859, Campinas, São Paulo, Brazil.*

‡*University of Brasília, Faculty UnB Planaltina, Materials Science Postgraduate Program, Brasília, Federal District, Brazil.*

¶*Center for Agri-food Science and Technology, Federal University of Campina Grande, 58840-000, Pombal, Paraíba, Brazil.*

§*University of Brasília, Institute of Physics, Physics Postgraduate Program, 70910900, Brasília, Federal District, Brazil.*

‖*Institute of Physics and International Center of Physics, University of Brasília, 70910-900, Brasília, Federal District, Brazil.*

⊥*Institute of Physics, University of Brasília, 70910-900, Brasília, Federal District, Brazil.*

#*Computational Materials Laboratory, University of Brasília, 70910900, Brasília, Federal District, Brazil.*

@*University of Brasília, College of Technology, Department of Electrical Engineering, 70910-900, Brasília, Federal District, Brazil.*

△*Materials Science and NanoEngineering Department, Rice University, 77005, Houston, Texas, United States.*

E-mail: marcelo.lopes@unb.br

Phonon dispersion and dynamic stability

To evaluate the dynamic stability of pristine and porous 12-AGNRs, phonon dispersion relations were computed using density functional perturbation theory (DFPT). Figure S2 displays the phonon spectra along the high-symmetry Γ–X path of the Brillouin zone. The insets provide a detailed view of the low-frequency region, where the absence of imaginary modes confirms the stability of both structures.

The phonon dispersions of the porous nanoribbons exhibit slightly broader distributions in the low-frequency range due to the increased degrees of freedom introduced by periodic porosity. However, no soft modes are observed, ensuring that both configurations maintain structural integrity at zero temperature. The presence of high-frequency modes around 3200 cm^{-1}, associated with C–H bond stretching vibrations, remains unchanged, further indicating that hydrogen passivation effectively stabilizes the pore edges.

These results validate the structural robustness of the investigated nanoribbons, reinforcing their suitability for nanoelectronic and optoelectronic applications.

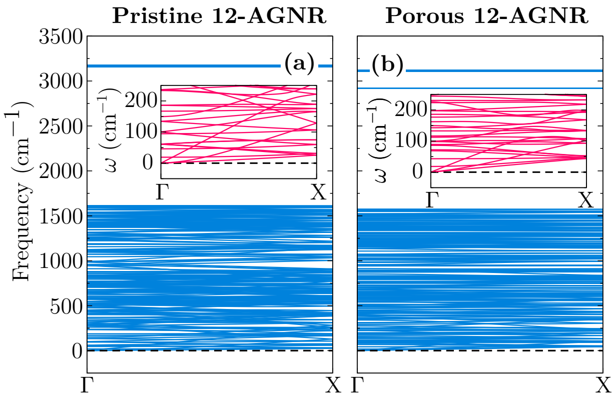

Figure S1: Phonon dispersion relations of pristine (a) and porous (b) 12-AGNRs. Insets provide a zoomed-in view of the low-frequency region, highlighting the absence of imaginary modes, which confirms the dynamic stability of both structures.

Thermal stability analysis via AIMD simulations

To further assess the thermal stability of pristine and porous 12-AGNRs, we performed *ab initio* molecular dynamics (AIMD) simulations at 300 K and 1000 K within the canonical (NVT) ensemble. Figure S1 presents the energy per atom as a function of time for each system. The insets show the corresponding temperature fluctuations and the atomic structures (top and side views) at the end of the simulations.

At both temperatures, the systems exhibit stable energy profiles with no significant structural distortions, reinforcing the robustness of the nanoribbons. The temperature fluctuations remain within expected ranges, indicating an efficient thermal equilibrium. Additionally, no bond-breaking or major reconstructions were observed, further confirming the thermal stability of the investigated structures. These results support the validity of our computational framework and align with experimental findings, demonstrating that both pristine and porous 12-AGNRs retain their structural integrity under ambient and high-temperature conditions.

Bethe-Salpeter Formalism for Excitons

In this work, the excitonic states are obtained by the solution of solving the BSE[S1] using the Tamm-Dancoff approximation (TDA).[S2] The excitonic Hamiltonian H_{exc}, is composed by the electron H_e and hole H_h, single particle Hamiltonians plus Coulomb interaction potential V_{eh}, which binds the electron-hole pairs, i.e,

$$H_{\text{exc}} = H_e + H_h + V_{eh}. \tag{1}$$

The single particle Hamiltonian (H) and overlap matrices (S), are written as the following:

$$H(\mathbf{k}) = H(\mathbf{R_0}) + \sum_{i}^{N} e^{i\mathbf{k}.\mathbf{R_i}} H(\mathbf{R_i}), \tag{2}$$

and

$$S(\mathbf{k}) = S(\mathbf{R_0}) + \sum_{i}^{N} e^{i\mathbf{k}.\mathbf{R_i}} S(R_i), \tag{3}$$

where $H(\mathbf{R_0})$ and $S(\mathbf{R_0})$ are the Hamiltonian and overlap matrix of the unit cell respectively, $H(\mathbf{R_i})$ is the interaction Hamiltonian term of the unit cell with the neighbor unit cell at $\mathbf{R_i}$, $S(\mathbf{R_i})$ is the equivalent for the overlap matrix, all of these matrices are directly obtained from HONPAS using SISL package.[S3] Those single-particle eigenvalues are obtained as the following:

$$H(\mathbf{k})\ |i, \mathbf{k}\rangle = E_{i,\mathbf{k}}\ S(\mathbf{k})\ |i, \mathbf{k}\rangle, \tag{4}$$

where i are band index respectively, $E_{i,\mathbf{k}}$ and $|i, \mathbf{k}\rangle$ are the eigenvalues and eigenvectors, respectively, $\mathbf{k}$ are the $\mathbf{k}$-point in BZ. It's also important to remember that electron and hole states are obtained by the same equation 4, and the difference between an electron and a hole state is their occupation.

The excitonic states with momentum center of mass $\mathbf{Q}$ are defined in terms of the product of electron- and hole-pair wave functions, as fol-

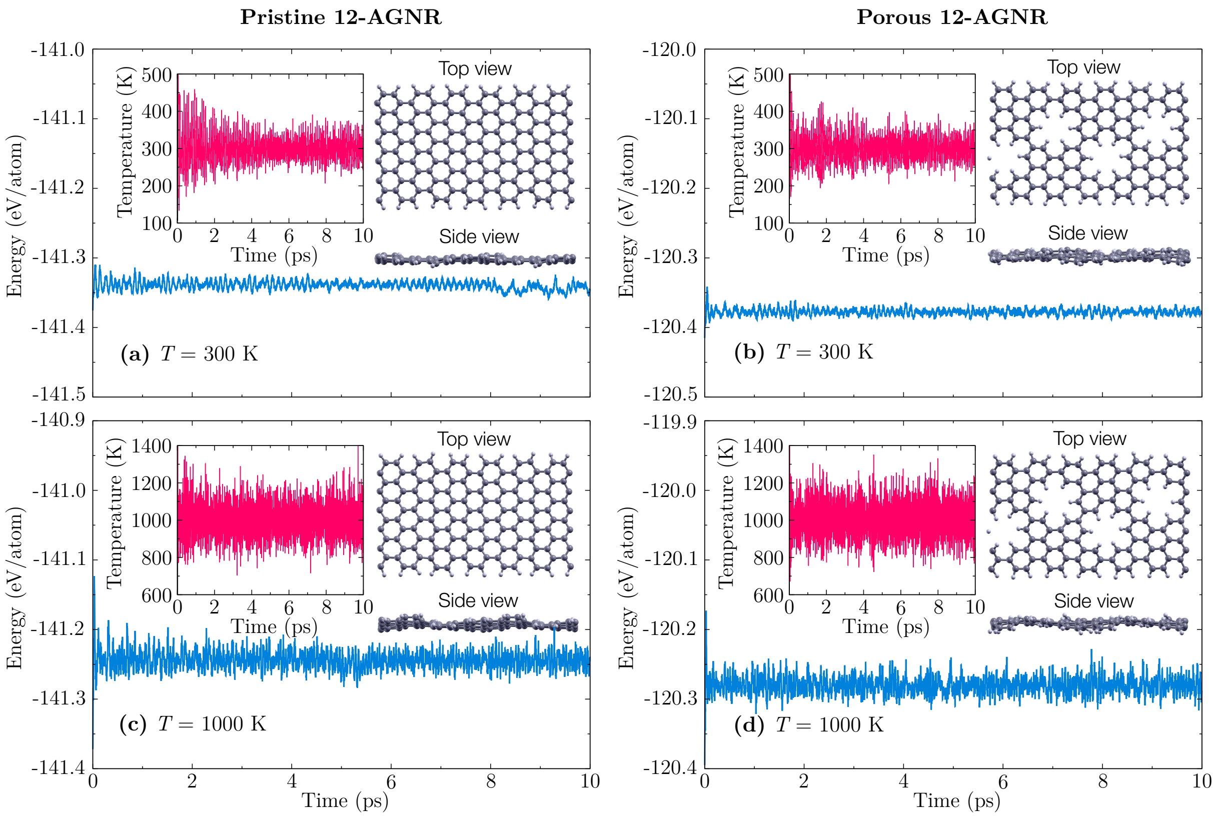

Figure S2: Energy per atom as a function of time for pristine (left) and porous (right) 12-AGNRs at 300 K (top) and 1000 K (bottom), obtained from *ab initio* molecular dynamics simulations. Insets show the temperature fluctuation over time and atomic configurations (top and side views) at the end of the simulations.

lows,

$$\Psi^n_{ex}(\mathbf{Q}) = \sum_{c,v,\mathbf{k}} A^n_{c,v,\mathbf{k},\mathbf{Q}} \ \left(|c, \mathbf{k}+\mathbf{Q}\rangle \otimes |v, \mathbf{k}\rangle\right), \tag{5}$$

where the index c e v corresponds to the conduction and valence bands states, with momentum $\mathbf{k}+\mathbf{Q}$ and $\mathbf{k}$, respectively.

The BSE eigenvalue equation,[S1] are obtained using the expansion of equation 5, as the following

$$\begin{aligned}&(E_{c,\mathbf{k}+\mathbf{Q}} - E_{v,\mathbf{k}})\, A^n_{c,v,\mathbf{k},\mathbf{Q}} \\ &+ \frac{1}{N_k} \sum_{\mathbf{k}',v',c'} W_{(\mathbf{k},v,c),(\mathbf{k}',v',c'),\mathbf{Q}} \ A^n_{c',v',\mathbf{k}',\mathbf{Q}} \\ &= E^n_{\mathbf{Q}} A^n_{c,v,\mathbf{k},\mathbf{Q}},\end{aligned} \tag{6}$$

where N_k is the number of $\mathbf{k}$-points in the BZ, $E^n_{\mathbf{Q}}$ are the energy of n-th excitonic state with momentum $\mathbf{Q}$, $A^n_{c,v,\mathbf{k},\mathbf{Q}}$ are the exciton wave function (eigenvector), which are obtained by solving equation 6. $E_{c,\mathbf{k}+\mathbf{Q}} - E_{v,\mathbf{k}}$ are the single-particle energy difference between a conduction band state c with momentum $\mathbf{k}+\mathbf{Q}$ and a valence band state v with momentum $\mathbf{k}$, and $W_{(\mathbf{k},v,c),(\mathbf{k}',v',c'),\mathbf{Q}}$ are the many-body Coulomb interaction matrix element, which can be divided into two parts, direct interaction, W^d, and exchange interaction, W^x, respectively, i.e.,

$$\begin{aligned}&W_{(\mathbf{k},v,c),(\mathbf{k}',v',c'),\mathbf{Q}} = \\ &W^d_{(\mathbf{k},v,c),(\mathbf{k}',v',c'),\mathbf{Q}} + W^x_{(\mathbf{k},v,c),(\mathbf{k}',v',c'),\mathbf{Q}}.\end{aligned} \tag{7}$$

Since the Coulomb potential varies slightly inside unit cell in comparison with Bloch functions, we can approximate the orbital character of the Coulomb term for non-orthogonal electron and hole single particle Hamiltonians by

the following approximation[S4]

$$W^d_{(\mathbf{k},v,c),(\mathbf{k}',v',c'),\mathbf{Q}} = V(\mathbf{k}-\mathbf{k}')\times \left\langle c,\mathbf{k}+\mathbf{Q}\left|\frac{1}{2}\left(S(\mathbf{k}+\mathbf{Q})+S(\mathbf{k}'+\mathbf{Q})\right)\right|c',\mathbf{k}'+\mathbf{Q}\right\rangle \times \left\langle v',\mathbf{k}'\left|\frac{1}{2}\left(S(\mathbf{k}')+S(\mathbf{k})\right)\right|v,\mathbf{k}\right\rangle \quad (8)$$

and

$$W^x_{(\mathbf{k},v,c),(\mathbf{k}',v',c'),\mathbf{Q}} = -V(\mathbf{Q}) \times \left\langle c,\mathbf{k}+\mathbf{Q}\left|\frac{1}{2}\left(S(\mathbf{k}+\mathbf{Q})+S(\mathbf{k})\right)\right|v,\mathbf{k}\right\rangle \times \left\langle v',\mathbf{k}'\left|\frac{1}{2}\left(S(\mathbf{k}')+S(\mathbf{k}'+\mathbf{Q})\right)\right|c',\mathbf{k}'+\mathbf{Q}\right\rangle . \quad (9)$$

In BSE formalism, the real and imaginary parts of the frequency dependent dielectric tensor $\epsilon^{BSE}_{1,\alpha,\beta}(\omega)$ and $\epsilon^{BSE}_{2,\alpha,\beta}(\omega)$ are obtained from the following expressions:

$$\epsilon^{BSE}_{1,\alpha,\beta}(\omega) = \delta_{\alpha,\beta} + \frac{e^2 S_p}{\epsilon_0 \Omega N_{\mathbf{k}}} \sum_n F^{n,BSE}_{\alpha,\beta} \frac{E^n_0 - \hbar\omega}{(\hbar\omega - E^n_0)^2 + \eta^2} , \quad (10)$$

$$\epsilon^{BSE}_{2,\alpha,\beta}(\omega) = \frac{e^2 S_p}{\epsilon_0 \Omega N_{\mathbf{k}}} \sum_n F^{n,BSE}_{\alpha,\beta} \frac{\eta}{(\hbar\omega - E^n_0)^2 + \eta^2} , \quad (11)$$

where E^n_0 is the n-th direct ($\mathbf{Q} = 0$) excitonic state energy, $F^{n,BSE}_{\alpha,\beta}$ is the excitonic modulated oscillator force, given by,

$$F^{n,BSE}_{\alpha,\beta} = \left(\sum_{c,v,\mathbf{k}} \frac{A^n_{c,v,\mathbf{k},0}\langle c,\mathbf{k}|P_\alpha|v,\mathbf{k}\rangle}{(E_{c,\mathbf{k}} - E_{v,\mathbf{k}} + i\eta_1)}\right) \times \left(\sum_{c',v',\mathbf{k}'} \frac{A^{n*}_{c',v',\mathbf{k}',0}\langle v',\mathbf{k}'|P_\beta|c',\mathbf{k}'\rangle}{(E_{c',\mathbf{k}'} - E_{v',\mathbf{k}'} - i\eta_1)}\right) . \quad (12)$$

Optical Properties

To obtain the optical properties, we calculated the real and imaginary parts of the frequency dependent dielectric tensor, $\epsilon_{1,\alpha,\beta}(\omega)$ and $\epsilon_{2,\alpha,\beta}(\omega)$, respectively, through the following expressions:

$$\epsilon_{1,\alpha,\beta}(\omega) = \delta_{\alpha,\beta} + \frac{e^2 S_p}{\epsilon_0 \Omega N_{\mathbf{k}}} \sum_{\mathbf{k},c,v} F^{c,v,\mathbf{k}}_{\alpha,\beta} \frac{(E_{c,\mathbf{k}} - E_{v,\mathbf{k}}) - \hbar\omega}{(\hbar\omega - (E_{c,\mathbf{k}} - E_{v,\mathbf{k}}))^2 + \eta^2} , \quad (13)$$

$$\epsilon_{2,\alpha,\beta}(\omega) = \frac{e^2 S_p}{\epsilon_0 \Omega N_{\mathbf{k}}} \sum_{\mathbf{k},c,v} F^{c,v,\mathbf{k}}_{\alpha,\beta} \frac{\eta}{(\hbar\omega - (E_{c,\mathbf{k}} - E_{v,\mathbf{k}}))^2 + \eta^2} , \quad (14)$$

where $\delta_{\alpha,\beta}$ is a delta kroenecker, S_p is the spin factor, being 1 for SOC and spin polarized calculations and 2 for non-polarized calculations, $F^{c,v,\mathbf{k}}_{\alpha,\beta}$, in the scope of independent particle approximation (IPA), corresponds to the oscillator force, defined by:

$$F^{c,v,\mathbf{k}}_{\alpha,\beta} = \frac{\langle c,\mathbf{k}|P_\alpha|v,\mathbf{k}\rangle\langle v,\mathbf{k}|P_\beta|c,\mathbf{k}\rangle}{(E_{c,\mathbf{k}} - E_{v,\mathbf{k}} - i\eta)(E_{c,\mathbf{k}} - E_{v,\mathbf{k}} + i\eta)} , \quad (15)$$

where Ω is the volume of unit cell, ϵ_0 is the vacuum permittivity constant, $N_{\mathbf{k}}$ is the number of $\mathbf{k}$-points employed for the BZ integration, ω is the incident photon frequency, $c(v)$ corresponds to the conduction(valence) states. η is a parameter to smooth the dielectric function, α and β corresponds to the components x, y and z in the dielectric tensor. P_α corresponds to the light-matter interaction operator, which is given by,

$$P_\alpha = \frac{\partial H(\mathbf{k})}{\partial k_\alpha} , \quad (16)$$

where $H(\mathbf{k})$ corresponds to the electronic Hamiltonian, and $\alpha = x, y, z$. For circularly polarized light, selected by the flag `CPOL= T`, the light-matter interaction operator for $\sigma_\pm$ are written by the following expression:

$$P_{\sigma\pm} = \frac{1}{\sqrt{2}} \left(\frac{\partial H(\mathbf{k})}{\partial k_x} \pm i \frac{\partial H(\mathbf{k})}{\partial k_y} \right) \ . \quad (17)$$

It's well known the relation between the complex dielectric constant $\epsilon(\omega)$ and the complex refractive index $\tilde{n}(\omega) = n(\omega) + i\kappa(\omega)$, where $n(\omega)$ is the refractive index and $\kappa(\omega)$ the extinction coefficient. They are related by:

$$\epsilon(\omega) = \tilde{n}^2(\omega) \ , \quad (18)$$

separating $\epsilon(\omega)$ in real and imaginary part, $\tilde{n}(\omega)$ in refractive index and extinction coefficient we have:

$$\epsilon_{1,\alpha,\beta}(\omega) + i\epsilon_{2,\alpha,\beta}(\omega) = n^2_{\alpha,\beta}(\omega) + 2in_{\alpha,\beta}(\omega)\kappa(\omega) - \kappa^2_{\alpha,\beta}(\omega) \ . \quad (19)$$

Then, we obtain the real and imaginary part of dielectric function, i.e.,

$$\epsilon_{1,\alpha,\beta}(\omega) = n^2_{\alpha,\beta}(\omega) - \kappa^2_{\alpha,\beta}(\omega) \ , \quad (20)$$

$$\epsilon_{2,\alpha,\beta}(\omega) = 2n_{\alpha,\beta}(\omega)\kappa_{\alpha,\beta}(\omega) \ . \quad (21)$$

From these two equations we derive the expressions of the extinction coefficient and of the refractive index, as follows,

$$\kappa_{\alpha,\beta}(\omega) = \left[\frac{\sqrt{\epsilon^2_{1,\alpha,\beta}(\omega) + \epsilon^2_{2,\alpha,\beta}(\omega)} - \epsilon_{1,\alpha,\beta}(\omega)}{2} \right]^{\frac{1}{2}} \quad (22)$$

$$n_{\alpha,\beta}(\omega) = \left[\frac{\sqrt{\epsilon^2_{1,\alpha,\beta}(\omega) + \epsilon^2_{2,\alpha,\beta}(\omega)} + \epsilon_{1,\alpha,\beta}(\omega)}{2} \right]^{\frac{1}{2}} \ . \quad (23)$$

The absorption coefficient $A_{\alpha,\beta}(\omega)$ is defined as:

$$A_{\alpha,\beta}(\omega) = \frac{2\kappa_{\alpha,\beta}(\omega)\omega}{c} = \frac{\sqrt{2}\omega}{c} \left[\sqrt{\epsilon^2_{1,\alpha,\beta}(\omega) + \epsilon^2_{2,\alpha,\beta}(\omega)} - \epsilon_{1,\alpha,\beta}(\omega) \right]^{\frac{1}{2}} \ . \quad (24)$$

where c is the light speed. The reflectivity $R_{\alpha,\beta}(\omega)$ is defined by the refractive index and extinction coefficient by the following expression:

$$R_{\alpha,\beta}(\omega) = \frac{(n_{\alpha,\beta}(\omega) - 1)^2 + \kappa^2_{\alpha,\beta}(\omega)}{(n_{\alpha,\beta}(\omega) + 1)^2 + \kappa^2_{\alpha,\beta}(\omega)} \ . \quad (25)$$

The energy loss function $L_{\alpha,\beta}(\omega)$ is defined as:

$$L_{\alpha,\beta}(\omega) = \frac{\epsilon_{2,\alpha,\beta}}{\epsilon^2_{1,\alpha,\beta} + \epsilon^2_{2,\alpha,\beta}} \ . \quad (26)$$

The dielectric response of pristine and porous 12-AGNRs reveals the significant role of excitonic effects in both systems. As shown in Figure S3, the BSE results introduce multiple sharp features in the imaginary part of the dielectric function ($\mathrm{Im}(\varepsilon)$), especially at energies below the onset of the IPA prediction, indicating the formation of strongly bound excitons. These effects are more pronounced in the pristine 12-AGNRs, which exhibit sharper and more intense peaks in the sub-eV and visible ranges. The porous counterpart, in contrast, shows a wider optical gap and less intense excitonic features due to symmetry reduction and electronic localization from the periodic voids.

In the real part ($\mathrm{Re}(\varepsilon)$), pristine nanoribbons present higher values at low energies, suggesting enhanced polarizability compared to their porous analogs. The spectral weight redistribution and reduced field screening in the porous system further reinforce the conclusion that porosity significantly affects graphene nanoribbons' light-matter interaction mechanisms.

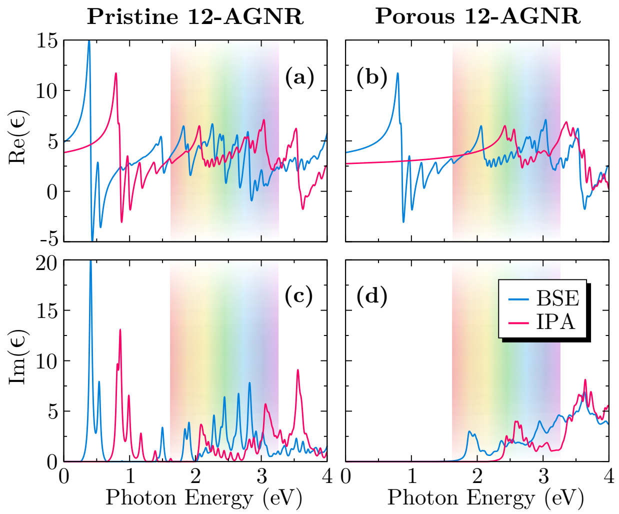

Figure S3: Real and imaginary parts of the dielectric function $\varepsilon(\omega)$ for pristine (left) and porous (right) 12-AGNRs computed using the Bethe–Salpeter equation (BSE, blue curves) and the independent-particle approximation (IPA, pink curves). Panels (a,b) show Re(ε) and (c,d) show Im(ε). The shaded area marks the visible spectrum.

Numerical Method for Effective Mass Calculation

Here, effective mass parameter is based on

$$\left(\frac{1}{m}\right)_{\mu\nu} = \frac{1}{\hbar^2}\frac{d^2\varepsilon}{dk^2} \tag{27}$$

so that its dimension is identified according to

$$\begin{aligned}\hbar^2 &\equiv \left(1.055\,457 \times 10^{-34}\mathrm{J\cdot s}\right)^2\\ &= (6.582\,119 \times 10^{-16})^2\mathrm{eV^2{\cdot}s^2}\\ &= \hbar_0^2\mathrm{eV^2{\cdot}s^2}\end{aligned} \tag{28}$$

On the other hand,

$$\frac{d^2\varepsilon}{dk^2} \equiv \frac{d^2\varepsilon_0}{dk^2}\left(\mathrm{eV}\cdot\mathrm{\mathring{A}}^2\right) \tag{29}$$

in order to

$$\begin{aligned}\left(\frac{1}{m}\right)_{\mu\nu} &= \frac{1}{\hbar_0^2(\mathrm{eV^2{\cdot}s^2})}\frac{d^2\varepsilon_0}{dk^2}(\mathrm{eV}\cdot\mathrm{\mathring{A}}^2)\\ &= \frac{1}{\hbar_0^2}\frac{d^2\varepsilon_0}{dk^2}\left(\frac{\mathrm{\mathring{A}}^2}{\mathrm{eV\cdot s^2}}\right)\end{aligned} \tag{30}$$

Consider that

$$\begin{aligned}\mathrm{eV} &= \left(1.602\,177 \times 10^{-19}\right)\mathrm{J} \equiv \mathrm{eV_0{\cdot}J},\\ \mathrm{J} &\equiv \frac{\mathrm{kg\cdot m^2}}{\mathrm{s^2}}\end{aligned} \tag{31}$$

Then, following that

$$\begin{aligned}\left(\frac{1}{m}\right)_{\mu\nu} &= \frac{1}{\hbar_0^2}\frac{d^2\varepsilon_0}{dk^2}\left(\frac{\mathrm{\mathring{A}}^2}{\mathrm{eV_0{\cdot}J\cdot s^2}}\right),\\ \mathrm{\mathring{A}}^2 &= 10^{-20}\mathrm{m}^2\end{aligned} \tag{32}$$

in which

$$\frac{\mathrm{\mathring{A}}^2}{\mathrm{eV_0{\cdot}J\cdot s^2}} = \frac{10^{-20}\mathrm{m}^2}{\mathrm{eV_0{\cdot}s^2}}\frac{\mathrm{s}^2}{\mathrm{kg\cdot m^2}} = \frac{10^{-20}}{\mathrm{eV_0{\cdot}kg}} \tag{33}$$

Therefore,

$$\begin{aligned}\left(\frac{1}{m}\right)_{\mu\nu} &= \frac{1}{\hbar_0^2}\frac{d^2\varepsilon_0}{dk^2}\left(\frac{10^{-20}}{\mathrm{eV_0{\cdot}kg}}\right) = \left(\frac{1}{m}\right)_{\mu\nu}\\ &= \frac{1}{\hbar_0^2}\frac{d^2\varepsilon_0}{dk^2}\left(\frac{10^{-20}}{1.602\,177 \times 10^{-19}{\cdot}\mathrm{kg}}\right)\end{aligned} \tag{34}$$

Including the $\hbar_0^2$ as a numerical constant

$$\begin{aligned}\left(\frac{1}{m}\right)_{\mu\nu} &= \frac{1}{(6.582\,119 \times 10^{-16})^2}\frac{d^2\varepsilon_0}{dk^2}\\ &\times\left(\frac{10^{-20}}{1.602\,177 \times 10^{-19}{\cdot}\mathrm{kg}}\right)\end{aligned} \tag{35}$$

$$\begin{aligned}\left(\frac{1}{m}\right)_{\mu\nu} &= \underbrace{\left[\frac{10^{31}}{(6.582\,119)^2 1.602\,177}\right]}_{C_1}\frac{d^2\varepsilon_0}{dk^2}\left(\mathrm{kg}^{-1}\right)\\ &= C_1\frac{d^2\varepsilon_0}{dk^2}\left(\mathrm{kg}^{-1}\right)\end{aligned} \tag{36}$$

So, according to $m = m_{\mathrm{eff}}\cdot m_0$, in which $m_0 = 9.109\,383 \times 10^{-31}$kg,

$$\frac{1}{m_{\mathrm{eff}}\cdot m_0} = C_1\frac{d^2\varepsilon_0}{dk^2}\left(\mathrm{kg}^{-1}\right) \tag{37}$$

thus,

$$\frac{1}{m_{\text{eff}}} = C_1 \cdot m_0 \cdot \frac{d^2\varepsilon_0}{dk^2}\left(\text{kg}^{-1}\right)$$
$$= C_1 \cdot \left(9.109\,383 \times 10^{-31}\text{kg}\right) \cdot \frac{d^2\varepsilon_0}{dk^2}\left(\text{kg}^{-1}\right) \tag{38}$$

from which

$$\frac{1}{m_{\text{eff}}} = \underbrace{\left[\frac{9.109\,383}{(6.582\,119)^2 1.602\,177}\right]}_{C_2} \frac{d^2\varepsilon_0}{dk^2} \tag{39}$$

and then

$$\frac{1}{m_{\text{eff}}} = C_2 \cdot \frac{d^2\varepsilon_0}{dk^2} \tag{40}$$

To calculate the derivative in 40, one consider that

$$\frac{d}{dk}\left[\frac{d\varepsilon_0}{dk}\right] = \frac{d}{dk}\left[\frac{\varepsilon_0(k+\Delta) - \varepsilon_0(k)}{\Delta}\right]$$
$$= \frac{1}{\Delta}\left[\frac{d}{dk}\varepsilon_0(k+\Delta) - \frac{d}{dk}\varepsilon_0(k)\right] \tag{41}$$
$$= \frac{1}{\Delta}\left[\frac{\varepsilon_0(k+2\Delta) - \varepsilon_0(k+\Delta)}{\Delta} - \frac{\varepsilon_0(k+\Delta) - \varepsilon_0(k)}{\Delta}\right]$$
$$= \frac{1}{\Delta^2}\left[\varepsilon_0(k+2\Delta) - 2\varepsilon_0(k+\Delta) + \varepsilon_0(k)\right]$$

Writing 40 through the tensor format,

$$\left(\frac{1}{m_{\text{eff}}}\right)_{\mu\nu} = C_2 \cdot \frac{d^2\varepsilon_0}{dk_\mu k_\nu} \tag{42}$$
$$= a_{\mu\nu} = \begin{pmatrix} a_{xx} & a_{xy} & a_{xz} \\ a_{yx} & a_{yy} & a_{yz} \\ a_{zx} & a_{zy} & a_{zz} \end{pmatrix} \tag{43}$$

Thus, to diagonal terms,

$$a_{\mu\mu} = \frac{1}{\Delta^2}\left[\varepsilon(k_\mu + 2\Delta) - 2\varepsilon(k_\mu + \Delta) + \varepsilon(k_\mu)\right] \tag{44}$$

whereas for the off-diagonal elements

$$a_{\mu\nu} = \frac{d\varepsilon}{dk_\mu}\left[\frac{d\varepsilon}{dk_\nu}\right] \tag{45}$$
$$= \frac{d\varepsilon}{dk_\mu}\left[\frac{\varepsilon(k_\mu, k_\nu + \Delta) - \varepsilon(k_\mu, k_\nu)}{\Delta}\right]$$
$$= \frac{1}{\Delta^2}[\varepsilon(k_\mu + \Delta, k_\nu + 2\Delta) - \varepsilon(k_\mu, k_\nu + \Delta) - \varepsilon(k_\mu + \Delta, k_\nu) + \varepsilon(k_\mu, k_\nu)]\ . \tag{46}$$

We obtained for the Pristine nanoribbons an effective mass of 0.0585 for the electron and 0.0581 for the hole, for the Porous nanoribbons we obtained 0.1516 for the electron and 0.1795 for the holes. As these materials are only periodic in $\hat{x}$ direction only the m_{xx} component of the effective mass was calculated.

Fracture Pattern

To analyze local stress concentrations and fracture mechanisms in pristine and porous 12-AGNRs, we computed the atomic von Mises stress σ_{VM}, which provides a scalar measure of local shear stress. This quantity is widely used to predict yielding and fracture initiation in solids under complex loading conditions. The atomic von Mises stress is defined as:

$$\sigma_{\text{VM}} = \left[\tfrac{1}{2}\big((\sigma_{xx} - \sigma_{yy})^2 + (\sigma_{yy} - \sigma_{zz})^2 + (\sigma_{zz} - \sigma_{xx})^2 + 6(\sigma_{xy}^2 + \sigma_{yz}^2 + \sigma_{zx}^2)\big)\right]^{1/2} \tag{47}$$

where σ_{ij} are the components of the local stress tensor at each atom.

Figure S4 illustrates the fracture morphology of pristine and porous nanoribbons under uniaxial tension. In both systems, fracture initiates in high-stress regions (in red) and propagates across the structure without significant plastic deformation, confirming a brittle failure mode. For pristine 12-AGNRs, fracture occurs at higher strains (∼37.7%) and is relatively uniform along the cross-section. In contrast, the porous structure fractures earlier (∼22.8%) and exhibits stress concentration near pore edges,

which serve as preferential sites for bond breaking and crack initiation.

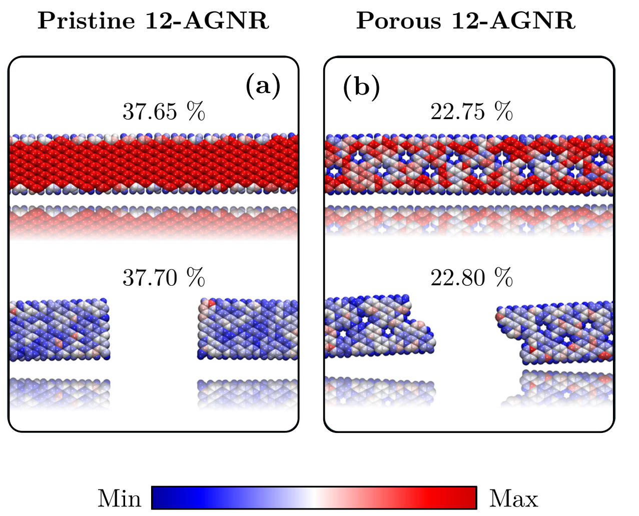

Figure S4: Fracture patterns of pristine (a) and porous (b) 12-AGNRs obtained from uniaxial tensile simulations. Color mapping corresponds to the atomic von Mises stress, with red indicating high local stress and blue indicating low-stress regions. The percentages indicate strain at the point of fracture propagation.

These findings highlight how periodic porosity weakens the carbon framework, reducing both the critical stress and strain and guiding fracture evolution through structurally defined stress amplifiers. This behavior emphasizes the importance of pore geometry in tuning mechanical robustness at the nanoscale.

References

(S1) Salpeter, E. E.; Bethe, H. A. A relativistic equation for bound-state problems. *Phys. Rev.* **1951**, *84*, 1232.

(S2) Alexander L. Fetter, J. D. W. *Quantum Theory of Many-Particle Systems*; Dover Publications, 2003.

(S3) Papior, N.; Febrer, P. sisl (v0.15.2). 2024; `https://doi.org/10.5281/zenodo.597181`.

(S4) Trolle, M. L.; Seifert, G.; Pedersen, T. G. Theory of excitonic second-harmonic generation in monolayer MoS_2. *Phys. Rev. B* **2014**, *89*, 235410.
